# Seasonal Bird Migration Could Explain Regional Synchronicity and Amplification in Human West Nile Virus Case Numbers

**DOI:** 10.1029/2024GH001194

**Published:** 2025-03-20

**Authors:** Franklin W. Schwartz, Motomu Ibaraki, Hiroko M. Hort

**Affiliations:** ^1^ School of Earth Science The Ohio State University Columbus OH USA; ^2^ GSI Environmental Inc. Irvine CA USA

**Keywords:** West Nile virus, migratory birds, terrestrial birds, northern great plains, cluster analysis, transmission pathway

## Abstract

West Nile virus (WNV) is a zoonotic virus with a mosquito‐avian transmission cycle having occasional spillover to mammals. A network analysis of annual log‐transformed WNV case numbers (2003–2022) generated four spatially and temporally coherent clusters among 48 U.S. states and six Canadian provinces. Cluster 1 and Cluster 3 were the largest groups corresponding to the Central Flyway and the closely associated Eastern Flyway (with an east‐coast subset). Cluster 2 and Cluster 4 corresponded with less‐well defined segments of a distinctly different Western Flyway. Thus, clustering can be explained by migratory pathways of terrestrial birds. We investigated avian involvement in the spread of WNV from potential sources in the southern U.S. Analyses revealed consistent patterns in log‐transformed case numbers of human WNV. This study highlights the significant role of migratory birds in shaping the spatiotemporal patterns of WNV incidence across North America. However, the observed variability in incidence also likely reflects the interplay of other factors including local environmental conditions, mosquito populations, and regional variations in both migratory and non‐migratory bird populations.

## Key Points

- Large annual incidences of human cases of West Nile virus (WNV) are associated with the Northern Great Plains of North America
- Statistical analyses implicate the spreading of WNV by terrestrial birds, such as blackbirds that migrate to southern states in fall
- The large WNV incidences represent a “perfect storm” in terms of large numbers of terrestrial birds and the most competent mosquito vector

## 1 Introduction

West Nile virus (WNV) is a zoonotic pathogen that cycles mostly between host species of birds and mosquitoes, with occasional spillovers to mammals, such as humans and horses (Bowen & Nemeth, 2007). However, of the >300 bird species known to have been infected with WNV (CDC, 2017), only a few are important for transmission of the virus (Kilpatrick et al., 2007). Birds are the principal wildlife reservoir hosts for the virus. Certain bird species also act as amplifying hosts for the virus (e.g., robins, sparrows, northern cardinals, common grackles, red‐winged blackbirds).

The severity of human infections caused by WNV varies. Approximately 80% of those infected will be asymptomatic. The remainder will experience a fever with “*other symptoms such as headache, body aches, joint pains, vomiting, diarrhea, or rash*” (CDC, 2023b) and recover. The risk of progressing to the more serious West Nile neuroinvasive disease is 1 in 150 cases, and among those with neuroinvasive disease, the mortality rate is 10% (Kaiser & Barrett, 2019). WNV was introduced to North America in 1999, and within 5 years, it spread across the continental United States (U.S.) and southern Canada.

From 1999 through 2022, in the U.S., the Centers for Disease Control and Prevention (CDC) reported 56,575 total cases with 2,776 fatalities (CDC, 2023a). Over the same period, there were 6,983 cases reported in Canada, with 150 deaths (Morshed, 2023; Public Health Agency of Canada, 2023). In Mexico, there were fewer than 20 reported cases of WNV (Elizondo‐Quiroga & Elizondo‐Quiroga, 2013).

Birds, the main host for WNV, were greatly affected by the initial spread of the virus across the continent after 1999 (Kilpatrick & Wheeler, 2019). American crows are extremely susceptible. In laboratory experiments, exposure to the virus resulted in a mortality rate approaching 100% (McLean, 2002). In the field, crow deaths were extraordinarily apparent (Caffrey et al., 2005) to the extent that mortality data through time proved useful in tracing the early spread of WNV (Kilpatrick & Wheeler, 2019). Agencies in the U.S. collectively tabulated 57,000 deaths of crows from 1999 to the end of 2002 (Caffrey et al., 2005). However, except for crows and a few other species, bird populations suffered no long‐lasting impacts (Kilpatrick & Wheeler, 2019).

Across North America, the distribution of four mosquito species in the *Culex* genus, that is, *Culex tarsalis, Culex pipiens, Culex quinquefasciatus and Culex restuans* can explain patterns of association with WNV cases (Rochlin et al., 2019). *Cx. tarsalis* is an important contributor to WNV transmission in western North America due to its high competence in spreading the virus to humans. Its abundance is closely linked to regions with the highest human risk (Rochlin et al., 2019). *Cx. tarsalis* mosquitoes favor an arid climate with hot and dry summers commonly associated with areas west of the Mississippi River in the U.S. and in western Canada (Rochlin et al., 2019). There is also an association with agricultural areas, particularly where irrigation creates small waterbodies (Reisen & Wheeler, 2019; Wimberly et al., 2008).

**Figure 1:**
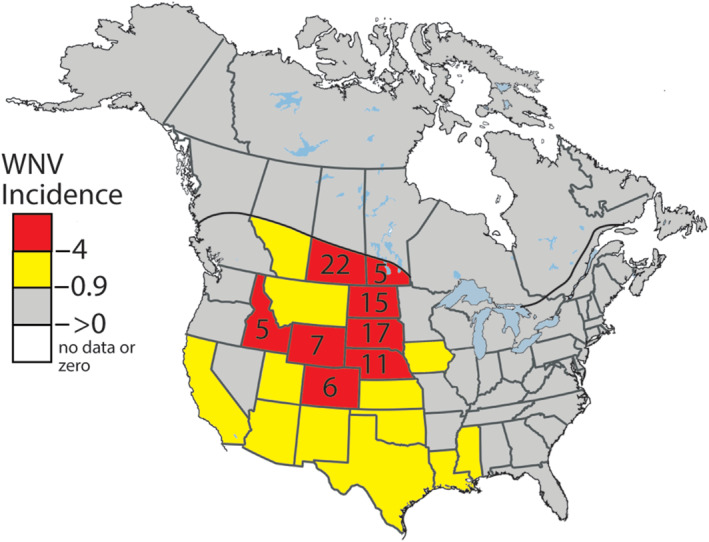
The mean annual incidence of human WNV per 100,000 people across the continental U.S. and populated areas of southern Canada from 2003 to 2022. For red areas (≥4), the number is the mean annual incidence of WNV per 100,000 people (British Columbia Center for Disease Control, 2023; CDC, 2023a; Government of Alberta, 2023; Government of Manitoba, 2023; Government of Quebec, 2023; Public Health Agency of Canada, 2023; Public Health Ontario, 2023; Statistics Canada, 2023; US Census Bureau, 2023).

*Cx. tarsalis* mosquitoes are abundant in the northern plains that is, North Dakota, South Dakota, and Nebraska, and parts of Prairie Canada. Average human WNV incidences from 2003 to 2022 (Figure 1) there were highest in North America. Elevated human incidences of WNV in Texas and Oklahoma Panhandles, western Kansas, and eastern Colorado, also coincided with conditions that are favored by *Cx. tarsalis* (Rey et al., 2012). However, in Texas and Oklahoma, *Cx. quinquefasciatus,* the dominant *Culex* species, drives transmission in urban areas, while *Cx. tarsalis* is more important in agricultural areas (Rochlin et al., 2019). The disease prevalence in these states could be lower because *Cx. quinquefasciatus* is considered a less competent human vector for WNV than *Cx. tarsalis* (Rochlin et al., 2019).

In the northeastern U.S., east of the Mississippi River, the dominant species, *Cx. pipiens* and *Cx. restuans* are indicated as the key vectors for WNV infections (Rochlin et al., 2019). In more rural areas, the average annual incidence of WNV is low because *Cx. pipiens* mosquitoes tend to affect bird populations more than humans, given that this species is ornithophagic. For example, average annual human incidence of WNV for Atlantic coastal states, Florida to Maine, was 0.2 cases per 100,000 people, ranging from 0.09 to 0.55. Our analyses of correlations of WNV cases between Florida and coastal Atlantic states suggested only modest spatial coherence in WNV case numbers from Florida northward. Thus, it is likely that reduced avian infections in the north, together with largely ornithophilic *Cx. pipiens and Cx. restuans* in the northeast have kept human WNV cases relatively low and uncorrelated.

In southern coastal states, such as Mississippi, Alabama, Georgia, Florida, and South Carolina, the mean annual incidences of WNV cases are low (Figure 1), with *Cx. quinquefasciatus* being the dominant mosquito vector (Rochlin et al., 2019). For those states east of the Mississippi River, the dominance of more ornithophagic species implies lower human incidences of WNV but continuing risks to birds (Rochlin et al., 2019).

Vector Control Districts or public health departments are at the forefront of innovative efforts to combat vector‐borne diseases, combining pioneering strategies—such as the use of genetically modified mosquitoes—with traditional methods like mosquito fogging, larvicides, and extreme habitat management (Chandra & Burman, 2024; OCMVCD, 2024). Progress in achieving long‐term solutions has been gradual, partly due to occasional surges in WNV cases observed in cities where cases were previously low (Kretschmer, 2023; Medina, 2024). Sustainable progress will require a deeper understanding of the environmental drivers that cause fluctuations in bird and mosquito populations, as well as a comprehensive exploration of how the virus is spread, transmitted, and amplified within these ecosystems. There remains a critical need for ongoing research and innovation to develop more effective, long‐lasting, and ecologically responsible solutions.

At the city or state scale, studies have shown that in a given year, the relative spatial variability in the incidence of human cases of WNV depends on (a) land use, (b) demographics, and (c) the relative abundance of known mosquito habitats, such as wetlands, agricultural or riparian settings, and (d) climate (DeGroote et al., 2008; Gibbs et al., 2006; Liu et al., 2008; Mori et al., 2018; Peterson et al., 2008; Ruiz et al., 2010; Winters et al., 2008). Figure 1 shows the geographic distribution of WNV across the U.S. and Canada as the mean annual incidence of WNV per 100,000 people from 2003 to 2022. The WNV incidence data for the extremely large Canadian provinces were applied to southern areas (Figure 1), which include almost all people, cities, and agricultural areas. The impacts of WNV are unevenly distributed, with an evident hotspot in the northern Midwest of the U.S. and southern Canada (Figure 1).

The hotspot includes six states and two provinces with mean annual WNV incidences ≥4. Southern Saskatchewan, North Dakota, South Dakota, and Nebraska exhibited mean annual incidences of ≥15 cases per 100,000 people from 2003 to 2022. Saskatchewan, North Dakota and South Dakota include the Prairie Pothole region, a vast area of small ponds and wetlands, which is a key nesting area for aquatic and terrestrial birds (Liu & Schwartz, 2012; McIntyre et al., 2019). Incidences in nearby states (yellow shading) and the Province of Manitoba were <4 and ≥0.9, respectively. Including areas with moderate WNV incidence (yellow), the major impacts of WNV, as indicated by the mean annual incidence, have been observed in the Midwest, east of the Rocky Mountains and states in the Southwest Region.

Studies at regional and national scales (Bowden et al., 2011; DeGroote & Sugumaran, 2012; Hahn et al., 2015a; Paull et al., 2017; Wimberly et al., 2008) have sought to elucidate the drivers of WNV primarily by examining the associations of WNV incidence rates with ecological and environmental factors, such as temperature, precipitation, and drought, as well as other controlling variables such as vegetation, host and vector prevalence, and public health controls. Findings have been generally inconsistent in identifying key variables. However, several studies, primarily at a local scale provided a level of predictability of either WNV infections or WNV vectors (Danforth et al., 2022; DeFelice et al., 2017; Little et al., 2016; Ward et al., 2023; Wimberly et al., 2022).

A major research question with WNV in North America is: How can the virus persist in places too cold for it to replicate (Reisen & Wheeler, 2019). Two strategies have been identified to answer this question: “reintroduction” and “persistence” (Reisen & Wheeler, 2019). Reintroduction would involve yearly re‐establishment of WNV in northerly areas with cold winters. One possible mechanism involves spreading by migratory birds moving north from overwintering areas, for example, in the southern U.S. or Central America.

There is a longstanding case for migratory birds being implicated as long‐distance spreaders of WNV, especially during the initial WNV spread away from New York City (Hadfield et al., 2019; Hort et al., 2023; McLean, 2002; Sullivan et al., 2006; Swetnam et al., 2018). This concept has been termed the “*migrant bird as introductory host theory*” (Rappole & Hubálek, 2003; Shelite et al., 2008). Human and nonhuman WNV cases were initially detected in New York City and surrounding areas in 1999 (Figure S1a in Supporting Information S1). Figures S1b–S1d in Supporting Information S1 depict spreading suggested spreading southward and westward in the U.S. By 2002, WNV had spread across the Midwest (Figure S1d in Supporting Information S1) (McLean, 2002). Once established in an area, arguments can be made as to overwintering mechanisms for WNV with mosquitoes in cold northern states/provinces or in warmer southern areas along with mosquitoes and overwintering migratory birds.

There have been concerns with ideas around avian spread of WNV in North America and elsewhere (Reisen & Wheeler, 2019). For example, local studies have indicated migrating birds were not carriers of WNV (Brown & O’Brien, 2011; Dusek et al., 2009). Other arguments against avian spread of WNV (Reisen & Wheeler, 2019; Shelite et al., 2008) were based on outdated ideas about bird migration behavior relevant to WNV. Terrestrial bird species, which spend most of their time on land, do not strictly follow Pacific, Central, Mississippi and Atlantic flyways, as do waterfowl, that is, ducks and geese (La Sorte et al., 2014).

WNV appears associated with migration patterns of terrestrial birds (Swetnam et al., 2018), which are loosely represented by Western, Central and Eastern Flyways (La Sorte et al., 2014). Boundaries of the Central Flyway overlap with the Eastern Flyway and change seasonally (La Sorte et al., 2014). Terrestrial birds returning south from nesting areas in the midwestern United States and Western Canada intermingle with birds from eastern parts of Canada and the U.S. in states bordering the Mississippi River (Dokter et al., 2018; Stonefish et al., 2021).

The migratory behaviors of terrestrial bird species commonly implicated as hosts for WNV, such as American robins (Kilpatrick et al., 2006), American crows (Hinton et al., 2015; Wheeler et al., 2014), and red‐winged blackbirds (Sullivan et al., 2006), are described by this three‐flyway model. The migratory behavior of some terrestrial species reflects great fidelity to their breeding areas but not to wintering grounds. With “partial migration” (Townsend et al., 2018), birds, such as crows, might migrate long distances, and other might remain nonmigratory residents of an area or only migrate shorter distances as necessary (Townsend et al., 2018).

Persistence involves mechanisms by which the virus locally endures long, cold winters or shorter cold snaps (Reisen & Wheeler, 2019). Diapause is a physiological state of arrested development that could enable infected mosquitoes to survive through northern winters. In the following spring, WNV can be transmitted to birds or other birds (Reisen & Wheeler, 2019). Quiescence is a strategy for mosquitoes to persist through short cold snaps. During quiescence, mosquito metabolism and development slow in response to cold temperatures (Tauber et al., 1986). Additionally, both laboratory and field data suggest that bird‐to‐bird transmission mechanisms could provide a persistence mechanism (Hinton et al., 2015; Komar et al., 2003; Reisen & Wheeler, 2019).

Other inferences about the spread of WNV have come from analyses of human WNV case numbers (Hort et al., 2023). Temporal and spatial synchronicity in annual log‐transformed case numbers extended along the Central Flyway from Texas through Oklahoma, Kansas, and Nebraska to the Dakotas, which included the hotspot area (Figure 1). Thus, relatively high annual values of the annual log‐transformed case numbers of WNV in Texas were associated with similarly high annual log‐transformed case numbers in these other states north of Texas.

Overall, this study aims to elucidate features of avian spread of WNV at a continental scale. The first objective was to expand analyses of WNV case correlations from seven states (Texas to North Dakota) to the continental U.S. and six Canadian provinces. Initial results suggested patterns of association in annual log‐transformed WNV case numbers or lack thereof should identify directions of spreading and suggest mechanisms for WNV persistence (Hort et al., 2023).

A second objective was to identify whether an illustrative bird species could be identified with regional migration characteristics leading to annual reintroduction of WNV. A phytogeographic approach found associations of WNV with migrating terrestrial birds and their importance in WNV spreading (Swetnam et al., 2018). A nuanced understanding of the pathways followed by terrestrial birds is important for better understanding avian dissemination of WNV and patterns in disease prevalence. For example, the preference of terrestrial birds for wetland and grassland settings of the Northern Great Plains and more specifically the Prairie Pothole Region has the potential to link features of the hydrologic setting to manifestations of WNV.

## 2 Methods

### 2.1 Data Sources

We utilized annual WNV data for counties and provinces in the U.S. and Canada, as reported by the CDC (CDC, 2023a), the Government of Canada's Surveillance of West Nile Virus Program (Public Health Agency of Canada, 2023), and various individual provincial websites in Canada (British Columbia Center for Disease Control, 2023; Government of Alberta, 2023; Government of Manitoba, 2023; Government of Quebec, 2023; Public Health Ontario, 2023). The population figures for both the U.S. and Canada were sourced from the U.S. Census Bureau and Statistics Canada (Statistics Canada, 2023; US Census Bureau, 2023).

This collection of data spanned years from 1999 to 2022 and encompassed the total number of human WNV disease cases, which included both neuroinvasive and non‐neuroinvasive cases. We chose to analyze data from 2003 onward, focusing on disease incidences occurring after WNV became endemic in the U.S. and Canada. The case numbers for Canada might differ somewhat from those in the U.S., given difference in medical practices. Although WNV arrived in the west coast states in 2003, the main outbreak there occurred in 2004. Elsewhere, the 2003 starting point for other states/provinces generally includes the highest case numbers for the U.S. and Canada. Thus, starting in 2003 may create a small outlier for the western states.

We report WNV in terms of total disease incidences in keeping with approaches followed our earlier study (Hort et al., 2023). There are limitations with the data and the use of total disease incidences instead of focusing on neuroinvasive cases, as discussed by McDonald (2021). The surveillance system likely underreports WNV incidence, especially for mild cases, and lacks comprehensive clinical data, leading to misclassification between neuroinvasive and non‐neuroinvasive forms. Additionally, the absence of confirmatory testing for WNV may result in false positives. Variations in disease awareness, testing capacity, and case definition changes over time also affect data accuracy, making comparisons across regions or time periods problematic, particularly with non‐neuroinvasive cases.

### 2.2 Network and Cluster Analysis

We used network and cluster analyses to describe the relationships between states and provinces in terms of log‐transformed annual case numbers of WNV. These methods have been applied across disciplines, including social studies, medicine, and earth science (Finn et al., 2014; Morrison et al., 2017; Zachary, 1977; Zitnik, 2014).

The calculated network was visualized using circles (nodes) and lines (edges) that connect circles. The nodes represent 48 continental states in the U.S. and six provinces in Canada (British Columbia, Alberta, Saskatchewan, Manitoba, Ontario, and Quebec). The distance between nodes in parts of the network yielded information on the strength of associations. Closer nodes were more closely related than were nodes farther away.

The Fruchterman‐Reingold layout algorithm facilitated the display of the network structure, along with graph sampling, to simplify the visualization (Fruchterman & Reingold, 1991). The Louvain method determined the optimal number of communities within the network, which was four. Thus, cluster analysis was a complimentary and unsupervised statistical approach for formally defining clusters (also termed communities) in an unbiased manner.

Preprocessing of the data involved two steps. We first created a data matrix using the log‐transformed annual WNV case numbers for the 54 states/provinces. This matrix was of the form *X* (*n*, *p*), where *n* was the number of states/provinces and *p* was the years with available WNV case data (Figure S2a in Supporting Information S1). Next, we calculated Pearson correlation coefficients (Ott & Longnecker, 2016) and their associated *r* values as a measure of the correlation between each pair of states/provinces. The resulting correlation matrix, *Y* (*n*, *n*), is depicted in Figure S2b of Supporting Information S1. Then, using this Pearson's *r* correlation matrix, a cosine similarity matrix was generated (Figure S2c in Supporting Information S1), where each element indicated the degree of similarity between all pairs of states and provinces.

We plot these correlation data to identify paired states/provinces exhibiting strong similarity. Finally, the network was formulated based on the cosine similarity matrix using the Fruchterman‐Reingold layout algorithm, and communities were assigned using the Louvain method (Figure S2d in Supporting Information S1). To note, our analysis is centered on pairwise comparisons to identify patterns of similarity between states, rather than groupwise comparisons, making multiple comparison adjustments unnecessary in this context.

All the statistical analyses were conducted using RStudio 2023.061.1 Build 524 in *R* version 4.3.1 (R Core Team, 2023). The Stats and ggcorrplot (Kassambara & Patil, 2023) packages were used, and figures were created using plotly (Sievert, 2019). Among the collection of software tools in igraph (Csárdi et al., 2024) in *R* was used for visualizing the similarity matrix.

### 2.3 Bird Species

We evaluated four bird species to determine their potential to facilitate spreading of WNV. Choices were evaluated in terms of four criteria: (a) bird populations are large and widely distributed across North America; (b) distribution of the breeding bird population should help explain the manifestation of human incidences in the Dakotas and Nebraska; (c) bird populations are migrating to the extent that the nonbreeding season would involve southern locations where WNV could remain endemic; and (d) patterns of migration could feasibly explain the spread of WNV in spring from southern sources to sinks as far north as Saskatchewan and northern states east of the Mississippi and vice versa in fall.

We relied on previous studies to evaluate terrestrial bird species in terms of their potential to spread WNV. The list includes the relatively abundant avian species commonly associated with WNV in the U.S., namely American robins and American Crows. However, given our focus on the area of high WNV incidence on the northern plains, we also considered bird species that are abundant in this region, as well as nationally. Selections were informed by seroprevalence testing of birds from the Red River Valley area of North Dakota and Minnesota. Of the 11 species of birds tested in 2004 and 2005, >80% (*n* = 195) of WNV indications were associated with four species, common grackles (*n* = 81), red‐winged blackbirds (*n* = 39), American robins (*n* = 32) and American crows (*n* = 12) (Bell et al., 2006).

American robins (*Turdus migratorius*) are among the most abundant terrestrial species in North America, with a population of several hundred million. They are extremely susceptible to infections with WNV and are considered to be a preferred host and superspreader across North America (Kilpatrick et al., 2006; Stein, 2011). Moreover, their preferred habitats often place robins near humans. American crows (*Corvus brachyrhynchos*) were selected because of the intimate association of crow mortality with the spread of WNV, especially in the early years (Caffrey et al., 2005).

Red‐winged blackbirds (*Agelaius phoeniceus*) are another common and numerous species, and associated with WNV. Sullivan et al. (2006) identified red‐winged blackbirds in North Dakota as “*an important viral dispersal mechanism with the ability to spread arborviruses such as WNV across the United States.*” They are competent WNV hosts, able to circulate high concentrations of the virus with minimal mortality (Komar et al., 2003; Sullivan et al., 2006). Common grackles (*Quiscalus quiscula*) are members of the blackbird family. They breed east of the Rocky Mountains across the northern plains. Their population numbers are lower than red‐winged blackbirds with a preference to areas with dwellings rather than wetlands (C. O. Nelms et al., 1994).

Necessary information for evaluating the distribution and migration patterns of these species came from data products available from eBird (Fink et al., 2023). Various abundance data, such as relative abundance and percentage of modeled seasonal populations, were downloaded for breeding and nonbreeding populations (Fink et al., 2023). Available eBird maps for these species provide the relative abundance of seasonal populations within North America during the breeding season (from 2008 to 2022) and were included as Supporting Information S1.

Our selection of bird species has not attempted to capture the diversity in bird species present in the Northern Great Plains in summer and winter. Seroprevalence testing in the Red River valley identified 11 species with WNV antibodies (Bell et al., 2006). Surveys in North Dakota found 160 bird species breeding there. The 50 most common species, except for house sparrows, either migrated short distances (*n* = 25) (i.e., north of the U.S./Mexico border) or long distances (*n* = 24) (Neotropical locations south of the border) (Igl & Johnson, 1995; Stewart & Kantrud, 1972).

### 2.4 Potential Biases

Citizen science data, for example, eBird, are known to suffer from inherent biases (Johnston et al., 2021). eBird has several features, such as a semistructured design using check lists, metadata providing information on the level of effort, and data filters, which reduce biases (Callaghan et al., 2021) but do not eliminate them (Da Silva et al., 2023).

## 3 Results

**Figure 2:**
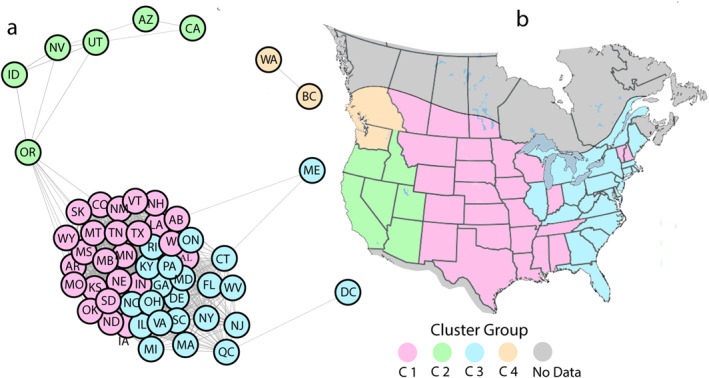
Network and cluster analyses of WNV human cases. The results of the network analysis of time series of annual log‐transformed human case numbers of WNV from 2003 to 2022 (a) yielded four cluster groups (C1 to C4). The spatial representation of these cluster groups on a map of North America (b) shows strong spatial coherence. For Canada, the coloring reflects the most populated southern areas. Places colored gray either have no cases or no data.

We used network and cluster analyses to describe the spatial and temporal coherence in human annual log‐transformed WNV case numbers among 48 states and six provinces. The calculated network (Figure 2a) showed 48 states, and six provinces organized on the basis of time series in their annual log‐transformed case numbers of WNV from 2003 to 2022. The nodes were organized as four clusters (C1 through C4). Most nodes were clustered in C1 and C3, in the central portion of Figure 2a. Geographically, cluster C1 included the provinces of Alberta, Saskatchewan, and Manitoba in Canada, the midwestern states east of the Rocky Mountains and several states east of the Mississippi River (Figure 2b). Cluster C3 encompassed Ontario and Quebec in Canada and more easterly states in the U.S., such as Michigan, Ohio, Illinois, and Kentucky (Figure 2b). Clusters C1 and C3, although somewhat differentiated geographically, are only subtlety different clusters with no obvious boundary between them. A smaller collection of coastal states, such as Connecticut, Massachusetts, New York, and Florida, were somewhat less strongly associated within C3. Maine and Washington DC, although assigned to C3 are poorly associated with the other states and provinces in C3.

Cluster C2 was a less well‐defined cluster (Figures 2a and 2b) containing states west of the Rocky Mountains. C4 included two nodes, Washington state and the province of British Columbia (Figures 2a and 2b). Taken together, the network analysis identified a Central Flyway (C1), a closely associated Eastern Flyway (C3) with a somewhat associated east‐coast collection of nodes. C2 and C4 were associated segments of a Western flyway, which was distinctly separated from the others.

We used multiple algorithms to optimize the approach to provide the “best” network. The maximum modularity (∼0.1) was obtained with the Louvain analysis. This value indicated a weak cluster structure, given that C2 and C4 were poorly defined and C1 and C3 were closely associated. Furthermore, we evaluated the results via silhouette analysis (Rousseeuw, 1987), which explored the goodness of fit of the algorithm for differentiating communities. The average silhouette width was 0.46, which was acceptable. Clusters C1 and C4 exhibited silhouette widths of approximately 0.7, where an average silhouette width over 0.7 was considered strong. Clusters C2 and C3 had lower silhouette widths (<0.4) and were low‐quality clusters. Network analysis (Figure 2a) also revealed that C3 was not distinct from C1. Based on this result, the states at the boundary of C1, such as WY, SK, and CO (Figure 2a), may not have been sharply differentiated in terms of their cluster structure.

**Figure 3:**
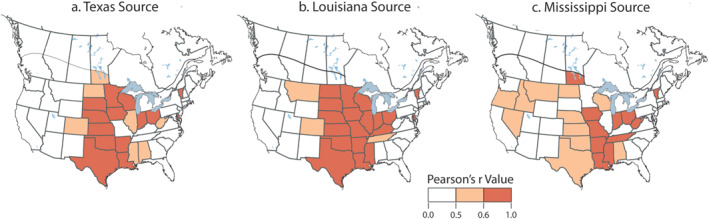
Associations of the time series of annual log‐transformed WNV case numbers for three potential source states represented as maps of Pearson's r values. With respect to Texas (a), there is a strong association in annual log‐transformed WNV case numbers northward as far as Minnesota and a moderate association with Manitoba and more easterly states. A potential source in Louisiana (b) has a strong association with many Midwest and Eastern States, including Vermont. Associations of WNV case data with Mississippi (c) are less pronounced than those with either Louisiana or Texas but include moderate associations with several states in the northwest.

Correlation results, as Pearson's *r* values, facilitated examinations and descriptions of correlations in the time series in annual log‐transforms of human WNV case numbers between Texas and 53 other states and provinces and similar comparisons using Louisiana and Mississippi. The resulting three maps (Figures 3a–3c) depicted strongly correlated case numbers, defined by Pearson *r* values ≥ +0.6, and somewhat correlated numbers with Pearson's *r* values between 0.5 and 0.6. The interpretation of correlation results followed typical practice with values in the range 0.00–0.19 as “very weak,” 0.20–0.39 as “weak,” 0.40–0.59 as “moderate,” 0.60–0.79 as “strong,” and 0.80–1.0 as “very strong.” To simplify plots and descriptions we considered Pearson's *r* values ≥ +0.6 to indicate a strong correlation and values between 0.5 and 0.6 to be somewhat correlated, a conservative lower limit (Owen, 2024).

The annual log‐transformed WNV case numbers for Texas were strongly correlated with those of nearby states, such as Oklahoma, Louisiana, and Arkansas (Figure 3a). Moreover, strong correlations also existed between Texas and states directly north to South Dakota, and modest correlations with North Dakota and Manitoba (Figure 3a). East of the Mississippi River, Indiana exhibited a strong correlation with Texas along with Vermont, Rhode Island, and Delaware. To better understand the correlations in Figure 3, examples of scatter plots have been added to Figure S3 in Supporting Information S1. They vary in terms of the goodness of correlation of annual log‐transformed WNV case numbers, that is, strong Pearson *r* values for South Dakota/Texas, Kansas/Texas, and Louisiana/Texas; moderate Pearson *r* values for North Dakota/Texas; and no correlation for Alabama/Louisiana (Figure S3 in Supporting Information S1).

Comparisons between Louisiana and other states and provinces showed extensive and continuous strong correlations in annual log‐transformed WNV case numbers northward to Minnesota and North Dakota and eastward to Kentucky, Ohio, and Vermont (Figure 3b). Compared to Texas, strong correlations were evident with states farther east, for example, Tennessee, Kentucky, and Ohio. The strong correlation in annual log‐transformed case numbers between Louisiana and Texas (Pearson's *r* +0.77) implied that midwestern states should have also been somewhat correlated with Texas. However, Pearson's *r* values, describing correlations in case numbers between Louisiana and Kansas, Nebraska, and South Dakota, were nearly identical to those of Texas, with North Dakota being significantly greater (compare Figures 3a and 3b). These results implied that events in Louisiana with respect to WNV also contributed to the strong correlations evident among these northern states.

Figure 3c shows the correlations between the state of Mississippi and other states/provinces. Because of the modest correlations of Mississippi with Texas (Pearson *r* +0.59) and Louisiana (Pearson's *r* +0.62), we expected somewhat weaker correlations with the Midwest states. Except for Missouri and Manitoba, the Pearson's *r* values associated with Mississippi were lower than those calculated for Louisiana. However, farther east, case numbers for Mississippi were strongly correlated with Tennessee (Pearson's *r* +0.80) and modestly correlated with West Virginia (Pearson's *r* +0.60). Thus, events in Mississippi had a modest influence on WNV case numbers in the Midwest but a stronger influence on Tennessee and West Virginia. There was also an evident correlation between the annual log‐transformed WNV case numbers in Mississippi and those in Idaho, Oregon, and Nevada (Figure 3c).

In Figure 3, Vermont exhibits a strong correlation with log‐transformed WNV case numbers in southern states, despite being isolated and geographically distant. However, with very few cases reported each year, mostly during high‐incidence years in the Midwest, the correlation may not be as robust, as it appears. Another possibility is apparent isolation due to bias in numbers for Pennsylvania and New York States with population centers in the east.

**Figure 4:**
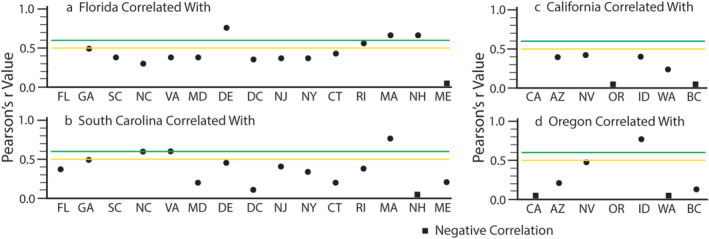
Pearson's r values representing correlations in log‐transformed WNV case numbers for Florida (a) and South Carolina (b) with eastern coastal states/districts. In the west, Pearsons r values depict correlations with California (c) and Oregon (d). The green and yellow lines represent the lower limit of correlations considered strong and modest, respectively. Negative correlations (*r* < 0) are indicated but not plotted.

Cluster C3 (Figure 2) also contained nodes representing states not correlated with Texas, Louisiana, or Mississippi. These were generally eastern states at or close to the Atlantic Ocean. We examined Florida and South Carolina in C3 to evaluate the spatial correlations along eastern coastal states (Figures 4a and 4b). In the case of Florida, nearby states were weakly to poorly correlated in terms of annual log‐transformed WNV case numbers. Only the Pearson *r* values for Delaware, Massachusetts, and New Hampshire were somewhat correlated (Figure 4a). The Pearson *r* values for South Carolina exhibited were modestly correlated with nearby states, Georgia, North Carolina, and Virginia (Figure 4b). Massachusetts was the only state exhibiting a strong correlation. In general, there were no obviously significant spatially coherent associations in terms of annual log‐transformed WNV case numbers.

The results were similar for the western states in C2 (Figure 2b). The Pearson *r* values for California, correlated with other western states (Figure 4c), indicated no correlation in terms of annual log‐transformed WNV case numbers. Oregon was strongly correlated with Idaho but none of the other states represented in Figure 4d.

The many Pearson correlation tests conducted here carry risks of Type I errors (biased parameter estimates). No adjustments were made to correct for Type I errors. The most important source of Type I errors was regression with independent variables that have associated errors (Brunner & Austin, 2007). The errors in tabulated WNV case numbers at this point are unknown but significant. A second source of Type I errors can be associated with problem of multiple testing–inferences derived from a single test may be significantly different than those derived from multiple scientific tests.

These correlation analyses suggested that the mechanisms involved in controlling the persistence of WNV in the Northern Great Plains and its connections to southern states were different from those operating in the eastern and western states on or along the two coasts.

The network analysis (Figure 2) and correlation analysis of human WNV case numbers (Figure 3) suggest an association with terrestrial migratory birds. We use population data from eBird to examine whether the hotspot for human WNV cases on the northern plains is associated with relatively large breeding populations of the four selected bird species (Fink et al., 2023). A second question is the extent to which migration in fall places a large non‐breeding population in southern states where overwintering of the virus could be favorable. Table 1 is created with population data from eBird for each state that summarizes relative proportion of each species (as a percentage) of the breeding (late spring and summer) and non‐breeding seasons (cold months, i.e., December through February) populations. The first area of interest (AOI‐1) includes North Dakota, South Dakota, and Nebraska. These states experienced the highest average annual incidence (2003–2022) of WNV in the U.S. The second area of interest (AOI‐2) includes Louisiana, Texas, Oklahoma, and Arkansas. These states offer possibilities as a source area of avian infection for northward migrating birds, maintained by infected birds overwintering or perhaps by endemicity of mosquitoes.

**Table 1:** Seasonal Distributions of Crows, Robins, Red‐Winged Blackbirds and Grackles in Canada, the U.S., and Mexico and Two Areas of Interest (AOIs)

Bird	Season	Populations (Percent)				
		Canada	USA	Mexico	AOI‐1	AOI‐2
American Crow	Breeding	36	64	0	2.9	13
	Non‐Breeding	20	80	0.1	2.2	12
American Robin	Breeding	46	53	1	5.0	0.7
	Non‐Breeding	0.9	98	2	6.7	17
Red‐winged	Breeding	23	74	3	15	13
Blackbird	Non‐Breeding	0	97	3	3.2	29
Grackle	Breeding	20	80	0	18	6.8
	Non‐Breeding	0	100	0	1	20

*Note.* Area population summaries are data products available from eBird Status and Trends (Fink et al., 2023). AOI‐1 includes North Dakota, South Dakota, and Nebraska. AOI‐2 includes Arkansas, Louisiana, Oklahoma, and Texas.

During the breeding season a significant proportion of the seasonal populations of robins and crows resided in Canada, less so with red‐winged blackbirds and grackles (Table 1). Moving to AOI‐1, the percentage of seasonal breeding populations of red‐winged blackbirds and grackles was much higher than with crows and robins (Table 1).

Because actual seasonal population sizes are different for the various species, relative abundance data provides another useful metric. Relative abundance is defined as “*count of individuals of a given species detected by an expert eBirder on a 1 hr, 2 km traveling checklist at the optimal time of day*” (Fink et al., 2023). For AOI‐1, during the breeding season the relative abundance varied: red‐winged blackbirds [8.0] > grackles [2.9] > robins [2.2] > crows [0.28]. Red‐winged blackbirds were substantially more abundant than others.

Although these four species are present in AOI‐2 during the breeding season (Table 1), our interest was with non‐breeding season following fall migration. Nearly 30% of the population of red‐winged blackbirds wintered in AOI‐2. Population percentages for grackles and robins were relatively high in AOI‐2 (Table 1). The data for crows for AOI‐1 and AOI‐2 suggested no obvious large‐scale migration to southern states to overwinter. Given these seasonal population numbers and migration behaviors, crows would contribute only locally to amplifying WNV in the breeding season and not to the avian spread of WNV with spring/fall migrations.

**Figure 5:**
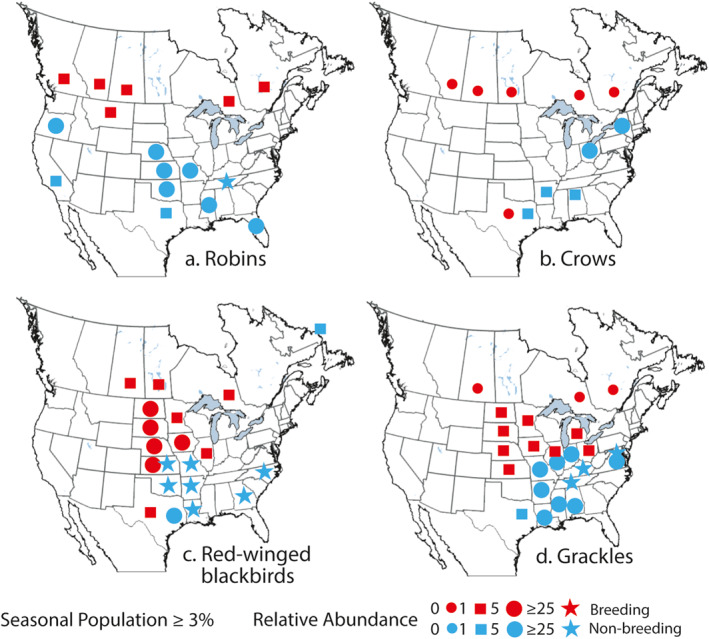
Maps depict the relative abundance of robins (a), crows (b), red‐winged blackbirds (c) and grackles (d) in places where the breeding, and nonbreeding populations of red‐winged blackbirds are greater than or equal to 3% of the seasonally modeled population. Population and abundance data were obtained from eBird (Fink et al., 2023).

Figure 5 provides a more general perspective on the seasonal population size and relative abundances of the four species of interest during breeding (red symbols) and non‐breeding seasons (blue symbols). For states/provinces with large populations of the four species, we show information on the relative abundance of species or counts of species a birder sees over a timed distance. We used a threshold of ≥3% of the modeled seasonal populations to identify states/provinces with the largest seasonal populations. The relative abundance of species is indicated by symbols in four classes. States/provinces without an added symbol indicate a seasonal population of less than 3%. During the breeding season, relative abundances of the four species were reduced in the U.S. because populations spread into Canada (Figure 5). However, in Canada, the relative abundances of these species were small.

The populations of robins and crows in the U.S. during the breeding season were mostly less than 3% except for Montana and Texas. There was no apparent preference for the northern plains (Figures 5a and 5b). A high resolution eBird visualization of relative abundance for robins (Figure S4 in Supporting Information S1) shows a breeding preference for areas around the Great Lakes and away from southern states. The visualization for crows (Figure S5 in Supporting Information S1) shows relatively small abundances in North Dakota through Kansas and states farther west, and in Canada. Crows were most abundant in southeastern states (Figure S5 in Supporting Information S1).

The breeding populations of both red‐winged blackbirds and grackles were greater than 3% in northern plains states (Figures 5c and 5d). eBird data summaries indicated relative abundances of red‐winged blackbirds between 5 and 10. Relative abundances of grackles were lower, typically from 1.8 to 3.3. The eBird visualization of relative abundances for red‐winged blackbirds (Figure S6 in Supporting Information S1) shows a strong association with the northern plain states, especially north and east of the Missouri River and extending into southeastern Saskatchewan in Canada. There is also some preference for areas around the Great Lakes. Abundances were relatively low in southeastern states. The pattern of relative abundances for Grackles is like red‐winged blackbirds (Figure S7 in Supporting Information S1). However, the considerably smaller population size resulted in lower relative abundances.

During the non‐breeding season, robins, red‐winged blackbirds, and grackles concentrate in warmer southern states (Figure 5). Thus, unlike population numbers for crows, this behavior is reflected by an increase in population numbers and relative abundances. Robins were less abundant in the plains states as compared to red‐winged blackbirds (Figures 5a and 5c). With red‐winged blackbirds, the largest populations and relative abundances are associated with states located along and west of the Mississippi River (Figure 5c). Values of relative abundance >25 are common, with values for Louisiana and Missouri of 137 and 115, respectively (Fink et al., 2023). Grackles tended to winter in states along and east of the Mississippi River (Figure 5d) with relative abundances in Tennessee and Kentucky of 52 and 25, respectively. Both red‐winged blackbirds and grackles are common along the Atlantic and Gulf coasts.

## 4 Discussion and Conclusions

The “*migrant bird as introductory host theory*” implies that in 2001, conditions were suitable for WNV, associated with infected birds migrating to southern states in fall, to persist over winter and to spread again during spring migrations. Our avian model assumes that WNV is reintroduced to northern areas each spring. The implication is that WNV has continued to persist in southern areas through the nonbreeding season (i.e., winter). Warmer temperatures in areas along the Gulf of Mexico likely maintain mosquito activity in winter (McLean, 2002; Tesh et al., 2004). Larger urban centers in the south might provide locally suitable settings for overwintering mosquitoes infected with WNV (Nasci et al., 2001). Other strategies might include facultative diapause or quiescence (Reisen & Wheeler, 2019), vertical transmission from an infected female to her F_1_ progeny (Goddard et al., 2003; B. M. Nelms et al., 2013) and bird‐to‐bird transmission within dense roosting communities, such as crows (Komar et al., 2003) and red‐winged blackbirds (Dolbeer & Linz, 2016). However, the evident correlation in the time series of annual log‐transformed case numbers of WNV with southern states implies that affected mosquitoes overwintering in cold northerly states are not a primary source of WNV.

Long distance migrants, that is, Neotropical birds, infected with WNV may also play a role in spreading WNV into southern states and further north. One example is the yellow‐headed blackbird, which is a member of the *Icteridae* family, along with red‐winged blackbirds. During the nonbreeding season, it occupies areas in central and coastal Mexico. During the breeding season, it is found exclusively in wetland/grassland areas of Saskatchewan, North Dakota, South Dakota, and Minnesota (Homan et al., 2004). Although yellow‐headed blackbirds are localized during the breeding season (Figure S8 in Supporting Information S1), the population numbers are large, approximately half those of red‐winged blackbirds. This species is not well studied with respect to WNV ecology. However, small numbers of infected birds were noted in North Dakota (Newbrey & Reed, 2008).

We profiled four species, American robins, American crows, red‐winged blackbirds and common grackles that might serve to spread WNV with migration. Red‐wing blackbirds provide the best fit with the four evaluation criteria. Their population is large and widely distributed, but with elevated numbers in the northern plains. They appear to be concentrated farthest south during the non‐breeding season, especially in Louisiana and Texas. These two states were identified by correlations in log‐transformed WNV case numbers as a potential starting point for avian spread.

Robins do not fit the criteria nearly as well. Their local breeding populations in North Dakota (1.3%) and South Dakota (1.5%) were relatively small. However, their status as a superspreader suggested that they could be an important contributor to seasonal magnification of WNV infections (Kilpatrick et al., 2006). When robins migrate from the northern plains, they could contribute to avian spreading of WNV. However, in the non‐breeding season, exposure of robins to WNV was likely minimal, given that the robin population was widely distributed, in the mid‐continent (e.g., Tennessee and Kentucky) with smaller relative abundances in Texas (4.6) and Louisiana (7.0). Crows are the worst fit with the criteria in terms of minimal breeding presence in the northern plains and tendencies toward local migration.

As noted, the wetlands and grasslands of the northern plains are breeding areas for a diverse and abundant collection of terrestrial migratory birds, other than those we examined. As is the case with robins, they likely contribute to magnification of WNV in summer. Their role as spreaders of WNV remains to be determined.

Population distributions and migration destinations for grackles are somewhat like red‐winged blackbirds (compare Figures 5c and 5d). However, their relatively smaller population and key wintering areas in Tennessee and Kentucky suggest they are less important in avian spread of WNV. However, huge roosts, which include these birds might facilitate bird‐to‐bird transmission during the non‐breeding season.

Given the possibility of warmer temperatures, red‐winged blackbirds in Louisiana could have a significant risk of acquiring WNV in winter. Correlation analysis (Figure 3) revealed that Louisiana was the principal avian source of WNV, followed by Texas. Mississippi was important as a source of eastward spreading. Moreover, large populations in Georgia (Figure 5c) could be a source of WNV in birds moving along the Atlantic coast in spring.

During the non‐breeding season, migratory terrestrial birds, which winter in Louisiana, Texas and other coastal states, later migrate to northern states, spreading the virus. The incidence of WNV in northern states (i.e., North and South Dakota, Nebraska) are usually much higher than the south but correlated to incidences in Texas (Hort et al., 2023). Resident birds in the northern and southern regions sustain local transmission, further contributing to WNV magnification during the breeding season. This dual dynamic underscores the role of both migratory and resident birds in the spread of WNV, with migratory birds serving as a bridge between distant locations, while local populations contribute to ongoing transmission.

We also calculated lagged correlation between Texas WNV caseloads (1 year lag) and those in northerly states as part of this study, However, correlations were moderate to weak (below *r* < 0.6), which further reinforced our finding that migratory birds contributed to the spread of WNV within the same year.

Red‐winged blackbirds, a wetland dweller, had the best potential to create the spatial and temporal correlations in WNV spreading patterns and contribute to the evident “hotspot” in WNV incidence in the Northern Great Plains area. Common grackles exhibited similar affinities to the northern plains; however, its smaller population and tendency to overwinter in areas farther north made it a less obvious choice.

Red‐winged blackbirds have been called the “most abundant passerine”, with a population of ∼250 million (Homan et al., 2004). Although their population is widely distributed across the continental U.S. and southern Canada during the breeding season, a sizable proportion, ∼15% of this seasonal breeding population, is in North Dakota, South Dakota, and Nebraska (Fink et al., 2023)^,^ with an additional 11.3% in Saskatchewan and Manitoba. Other blackbirds and robins co‐located in the states could contribute to local magnification of WNV. The populations of yellow‐headed blackbirds and common grackles are each approximately half that of red‐winged blackbirds (Homan et al., 2004). The size of the annual breeding population of *Icterids* (blackbirds) is more than 75 million in three states.

Minnesota, Iowa, and Illinois also host many red‐winged blackbirds during the breeding season (Figure 5c) but are associated with average annual incidence rates for WNV ranging between 0.7 and 1.0 per 100,000 people. These lower incidence rates are likely due to a small proportion of *Cx. tarsalis* mosquitoes that favor drier conditions farther west, and larger proportions of *Cx. pipiens* and *Cx. restuans*, considered to be strongly ornithophagic (Rochlin et al., 2019). For example, a study of mosquitoes associated with WNV in central Illinois found *Cx. pipiens* to be the dominant *Culex* species with *Cx. tarsalis* exhibiting mean abundances much less than 1% (Gardner et al., 2014). These states are at or distant from the eastern edge of the range of *Cx. tarsalis* (Rochlin et al., 2019).

The high human incidence of WNV associated with the Dakotas, Nebraska, and Canadian prairie provinces (Figure 1) appears to reflect a “perfect storm” in terms of the extraordinary size in the populations of avian hosts, and the presence of competent mosquito vectors. Remarkably, terrestrial birds, drawn to wetlands in the Prairie Pothole Region, co‐locate with large numbers of *Cx. tarsalis*, the most competent mosquito vector with respect to human transmission of WNV (Rochlin et al., 2019). Bloodmeal data for *Cx*. *tarsalis* indicate avian, mammalian, and human feeding tendencies (Rochlin et al., 2019). A study in California, found red‐winged blackbirds to be second in a list of avian hosts for *Cx. tarsalis* in a wetland setting (Thiemann et al., 2012).

In other parts of North America, human incidences of WNV are lower (Figure 1). Away from the Northern Great Plains, populations of breeding populations of red‐winged blackbirds and grackles are less concentrated (Figures S6 and S7 in Supporting Information S1). Areas to the east and southeast, outside of the range of *Cx. tarsalis* mosquitoes are commonly associated with ornithophagic mosquitoes and much lower WNV incidences. It is possible that the large amplification potential for WNV in the northern Plains region, given the large population of blackbirds and *Cx. tarsalis* mosquitoes, maintains the migration‐based spreading of WNV that began with the initial WNV introduction in 2002 (Figure S1d in Supporting Information S1).

Our interpretation of temporal and spatial coherence in log‐transformed case numbers as an indication of avian transport might seem inferential, simply based on the results of correlations in human WNV case numbers. However, the idea of spreading of WNV along bird migration routes is not solely based on correlation analyses but also on a variety of other data and observations from other studies (Hadfield et al., 2019; Moon et al., 2019; Swetnam et al., 2018). For example, the interpretation of avian migration pathways inferred from the correlations in Figure 3 is independently supported by migration patterns of red‐winged blackbirds (Figure 5) and grackles, which were inferred from eBird data and visualizations.

We briefly examine the possibility that other sources of spatial covariance in WNV case numbers might exist, which are unrelated to avian spreading. Weather and climate are well known to drive within‐year variability in human incidence of WNV, due to their associations with vector dynamics. For example, important mosquito traits, such as development rate, biting rate, adult lifespan, etc. vary significantly with temperature (Shocket et al., 2020). Human incidence of WNV peaked with an average summer temperature of 24°C (Shocket et al., 2020).

Studies have shown weather conditions to be associated with increased incidences of human WNV. Hahn et al. (2015b) found higher than normal incidences of neuroinvasive WNV disease associated with higher‐than‐normal temperatures and lower than average precipitation. Gorris et al. (2023) found seasonal association in human WNV incidence in areas with cold, dry winters, and wet and mild summers. Moreover, there was tendency for human acquired immunity to substantially reduce WNV incidence following a regional epidemic (Paull et al., 2017). In terms of the correlations in long‐distance, state‐level case numbers presented in this paper, weather‐related factors are important and can impact correlations (Hort et al., 2023).

Time varying WNV susceptibility in human and bird populations is another factor suggested as a potential source of covariance in WNV case numbers. Several features of our data would lead us to question this suggestion. Strong correlations in log transformed case numbers of human WNV are only evident in the Midwest. Susceptibility leading to covariance would be reasonably expected to occur elsewhere, such as the east and west coasts. The correlations have a directional aspect (Figure 3) with Louisiana as an obviously stronger source than either Texas or Mississippi. It would be difficult to explain these features in terms of susceptibility. More conventional banding studies of red‐winged blackbirds revealed that breeding birds from the Northern Great Plains wintered in large roosting communities in eastern Texas and Louisiana (Dolbeer, 1978, 1982). General confirmation of eBird results came from data on the migration pathways for red‐winged blackbirds in fall, which were interpreted from historical banding data (Dolbeer, 1978). These results also provided information on migration pathways and dispersion around those pathways.

**Figure 6:**
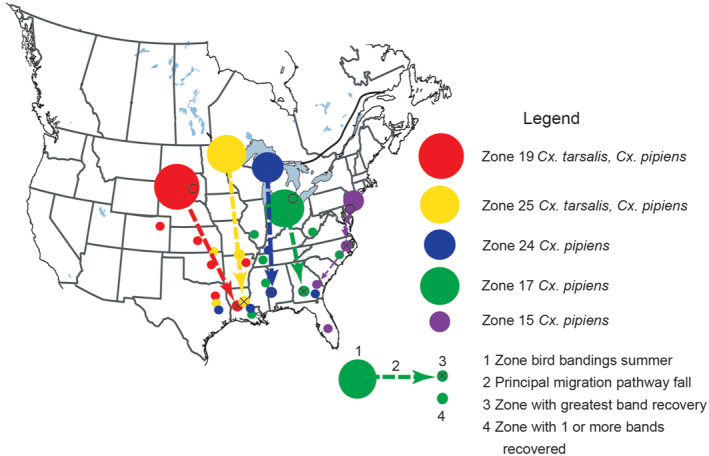
This figure shows primary, fall migration pathways for red‐winged blackbirds from five zones. Infections associated with *Cx. tarsalis* transmission in the Northern Great Plains possibly could be transported into Louisiana and East Texas with opportunities for dissemination to birds from Zone 17 and 24. Pathways were created based on data published by Dolbeer (1978).

Dolbeer (1978) subdivided the North American range of red‐wing black birds into 27 units and tabulated where some of the birds banded in each region during the breeding season were recovered during the winter roost period (10 December to 20 February). Figure 6 shows patterns of fall migration for red‐wing blackbirds that bred in five northern zones (Zones 25, 24, 19, 17, and 15). The data came from Dolbeer's Table 4. The diameter of circles in each of five zones was approximately equal to the width of the zone in the east‐west direction. The other smaller circles indicate zones where at least one of the bird bands was recovered. A migration pathway was defined for each these zones with the associated zone in winter roost period with the largest percentage of band recoveries.

The major pathways in the midcontinent (Figure 6) match those determined by correlations in log‐transformed WNV case numbers (Figure 3). Louisiana was the most important terminus for fall migrations. Moreover, the east‒west dispersion associated with terrestrial species is such that some fraction of red‐winged blackbirds from four northern areas co‐located in Texas and Louisiana. Similarly, birds from the lower Great Lakes Region wintered in the southern region from Louisiana to North Carolina (Figure 6). Along the Atlantic coast, birds from the Chesapeake Bay area wintered in more southerly coastal states as far south as Georgia (Dolbeer, 1978). Dispersion in this case also provided an opportunity for red‐winged blackbirds to co‐locate in winter with others from the midcontinent.

Hort et al. (2023) noted the amplification of WNV cases per 100,000 people relative to Texas, moving from Kansas to North Dakota. The magnitude of amplification of incidence rates increased nonlinearly as a function of incidence rates in Texas. We attributed this behavior to the presence of *Cx*. *tarsalis,* the most competent mosquito vector, and favorable grassland/agricultural/hydrologic settings where mosquitoes and birds are co‐located (Hort et al., 2023). The population of the proxy bird species, red‐winged blackbirds, is large there. Red‐winged blackbirds are present across southern Canada and the United States.

Large numbers of red‐winged blackbirds migrate southward to Louisiana, Texas, and Mississippi, where WNV appears capable of overwintering. Correlation analyses revealed that Louisiana was the most dominant potential source, with a large relative abundance of red‐winged blackbirds through the winter nonbreeding season. This species thus has population distributions and patterns of migration to serve in spreading WNV.

An obvious next step will involve examining other species of interest, such as the yellow‐headed blackbird. A focused assessment of the environmental conditions in southern coastal states related to overwintering is another step. We expect to discover climatic conditions in Texas and Louisiana southward to central Mexico during the nonbreeding season influencing the incidence of WNV cases. Progress in this respect should facilitate the prediction of the likelihood of human WNV cases in the northern Midwest and Prairie Canada during subsequent summers.

## Conflict of Interest

The authors declare no conflicts of interest relevant to this study.

## Supporting information

Supporting Information S1

## Data Availability

The data sets and code generated, analyzed, and visualized during this study are available in Zenodo. Hort, H. (2024). WNV_Data_v1.0 (WNV_Data). Zenodo. https://doi.org/10.5281/zenodo.13946679. The data for birds are publicly available from eBird (Fink et al., 2023) https://doi.org/10.2173/ebirdst.2022.
