# Supplementary material for: Seasonal Bird Migration Could Explain Regional Synchronicity and Amplification in Human West Nile Virus Case Numbers
Source: Geohealth. 2025 Mar 20;9(3):e2024GH001194. doi: 10.1029/2024GH001194 (PMC11923459; doi:10.1029/2024GH001194)
Supplement: Supplementary file 1 — Supporting Information S1 [file GH2-9-e2024GH001194-s001.pdf]

AGU GeoHealth

Supporting Information for

Seasonal Bird Migration Could Explain Regional Synchronicity and Amplification in Human West Nile Virus Case Numbers

Franklin W. Schwartz[1], Motomu Ibaraki[1], and Hiroko M. Hort[2*]

[1]School of Earth Science, The Ohio State University, Columbus, OH USA.

[2]GSI Environmental Inc., Irvine CA, USA.

*Corresponding author: hmhort@gsienv.com

Contents of this file

Text S1 to S8
Figures S1 to S8

Introduction

This supplementary information provides detailed insights into the methodologies, data analyses, and additional results that support the findings of our study on West Nile Virus (WNV) cases across North America. The figures included here elucidate various aspects of the network and cluster analyses, correlations in WNV case numbers, and the distribution patterns of specific avian species known to influence the spread of WNV.

Additional Information

This material uses data from the eBird Status and Trends Project at the Cornell Lab of Ornithology, eBird.org. Any opinions, findings, and conclusions or recommendations expressed in this material are those of the author(s) and do not necessarily reflect the views of the Cornell Lab of Ornithology.

Text S1.

One of the surprising features of the initial development of WNV in the U.S. was its rapid spread away from New York City in 1999. The progression on a county-by-county basis was captured by maps created by CDC, beginning in 1999 (Figure S1a) (Roehrig, 2013). By 2000, it had spread west to upstate New York and South to North Carolina (Figure 1b). The pace of WNV spread was best reflected in death of birds, which provided an early warning of human cases. Southward migration of infected birds in Fall 2000 set the stage for 2001 with progression of human and non-humans WNV cases south into Florida, west as far as Louisiana and Alabama in the south and Illinois and Wisconsin in the north (Figure 1c). By summer 2002, WNV had spread across the Midwest (Figure 1d).

Text S2.

Figure S2 describes the basic approach to network and cluster analyses.

Text S3.

Examples of the correlations in annual log-transformed WNV case numbers between the various states and provinces to obtain Pearson r values are shown in Figure S3. Strong correlations are evident with SD versus TX, KS versus TX, and LA versus TX. With an r value of 0.58, the correlation between ND and TX is weak, due to winter conditions in North Dakota in some years(Hort et al., 2023). The last example of AL versus LA is an example very weak correlation.

Text S4.

Figure S4 shows the distribution of robins (*Turdus migratorius*) across North America during breeding season. Breeding populations are spread widely from Mexico to Alaska and Arctic Canada. They are most abundant in the central Midwest and eastern states from Nebraska through Illinois, Ohio to New York State.

Text S5.

Figure S5 shows the distribution in abundance of American crows (*Corvus brachyrhynchos*) during the breeding season from 10 May to 5 July. Relevant to our study is the relatively small population of American crows in Kansas, Nebraska, South Dakota, North Dakota, and southern Saskatchewan. Human cases of WNV have been substantially elevated in these areas as compared to other areas.

Text S6.

Figure S6 shows the relative abundance of red-winged blackbirds (*Agelaius phoeniceus*). We selected this species as a proxy avian transmitter of WNV for three reasons. First, they are obviously associated with northern midwestern states and prairie provinces of Canada, given their significant abundance during the summer peak in WNV (Figure S6). Second, an early study of WNV in red-winged blackbirds (Sullivan et al., 2006) found substantial indications of WNV infection in the large breeding population of red-winged blackbirds in South Dakota. Thirdly, a significant proportion of this large population of birds winters in southern coastal states, where warmer temperatures possibilities for WNV to overwinter.

Text S7.

Common grackles represent another relatively large population of blackbirds that is found in Canada and the United States during breeding season (Figure S7). As the abundance map from eBird shows, the Northern Great Plains provides habitats favored by this species. The population in North Dakota is large, approximately the same as yellow-headed blackbirds.

Text S8.

In addition to red-winged blackbirds, other *Icterids* (blackbirds) could also play a role in avian spread of WNV. A relatively large breeding population of yellow-headed blackbirds (*Xanthocephalus xanthocephalus*) (Figure S8) is present mostly in the Northern Great Plains in states/provinces that are associated with the with the greatest incidences of human WNV cases. Unlike red-winged blackbirds, these birds winter much further south in coastal and central Mexico. In this area, it is likely that WNV would be endemic in bird and mosquito populations during non-breeding season.

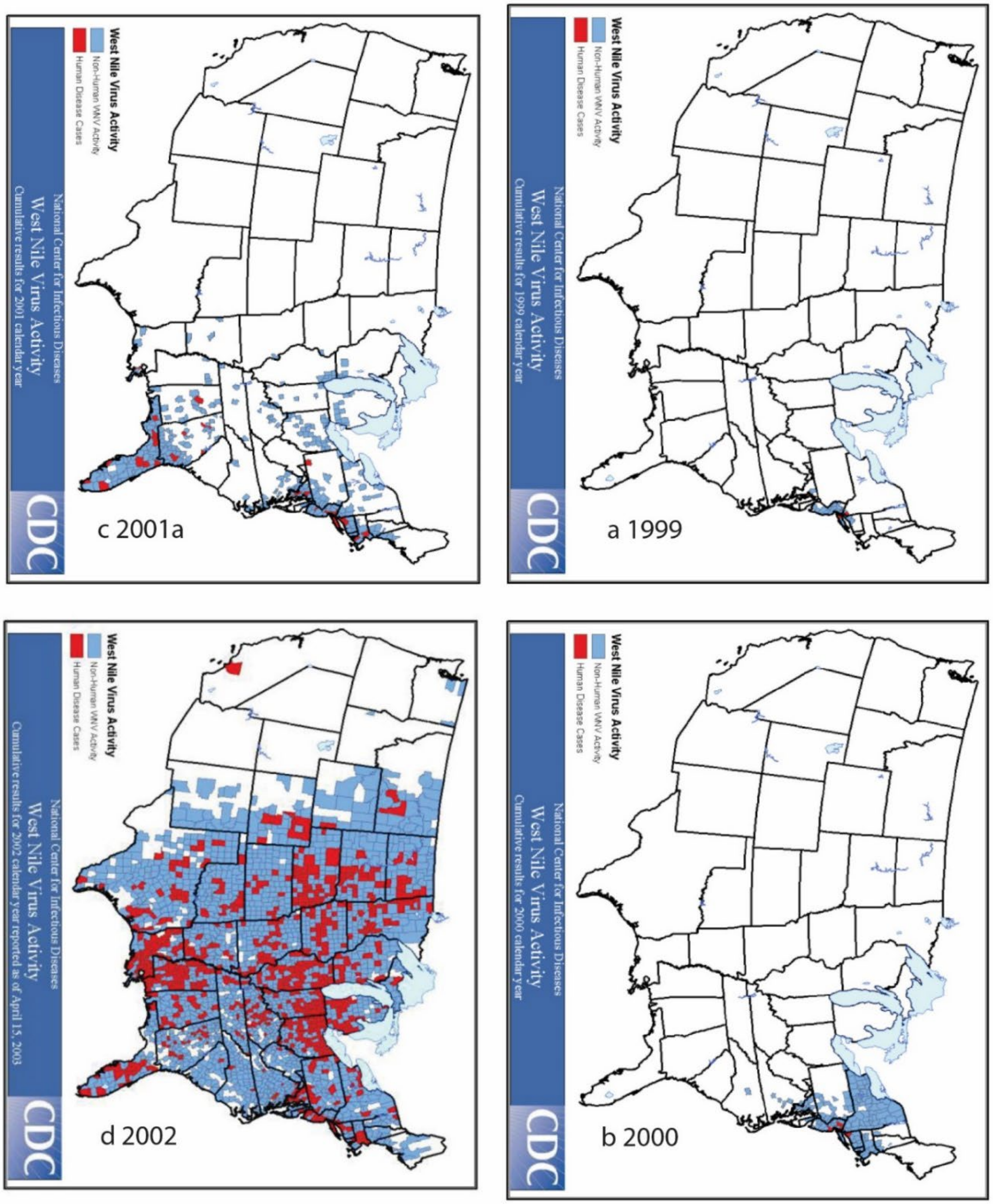

Figure S1. These maps originally published by CDC and assembled by Roehrig (2013) depict the annual progression in human (red) and non-human WNV cases (blue) in 1999 (a), 2000 (b), 2001 (c), and 2002 (d). (Public domain)

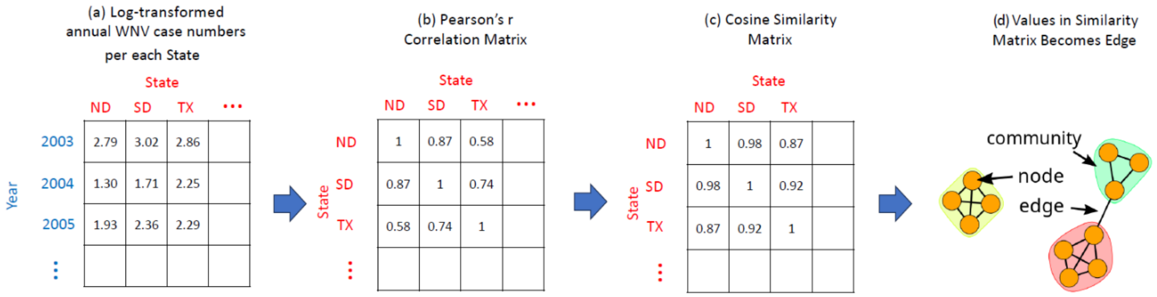

Figure S2. Steps in the network and cluster analyses. Panel (a) shows the general form of the data matrix, where the rows contain log-transformed annual WNV case numbers for the years 2003 to 2022. The matrix was the basis for estimating the Pearson's r correlation for each row in Panel (b). The cosine similarity was calculated to obtain the similarity matrix in Panel (c). Using the similarity matrix, igraph R software was used to visualize the network for each state and province, as shown in Panel (d). Once the communities were identified, the unique disposition of every state/province among the communities was known. Finally, we created maps of North America to describe whether geographical patterns among states and provinces existed.

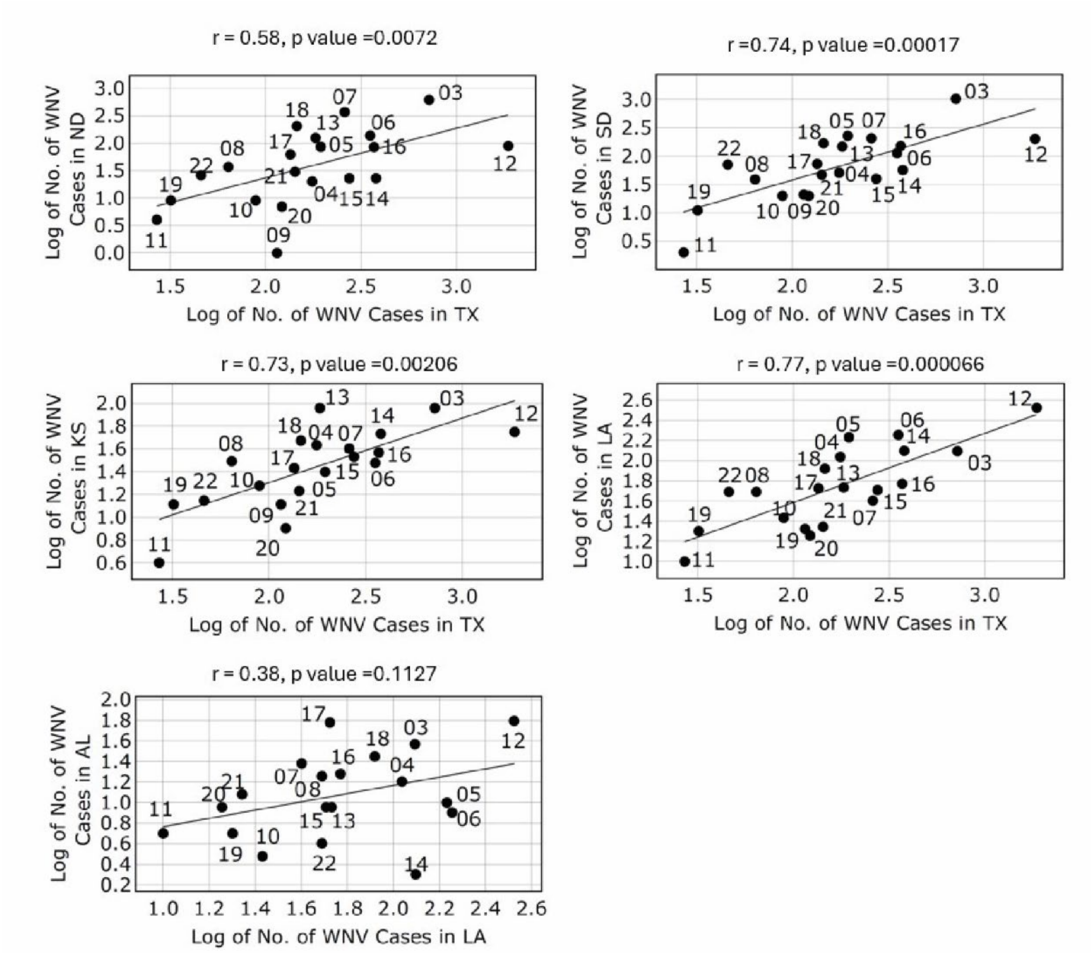

Figure S3. Five examples of the correlations in annual log-transformed case numbers between various states. With three of these examples, South Dakota, Kansas, and Louisiana, data plotted versus Texas represent strong correlations with r values >0.7. Data for ND versus TX suggests modest correlation. The poorest correlation is between Alabama and Louisiana with r = 0.38. An r value of 1.0 is perfect. The numbers xx next to the data points are years 20xx, for example 12 =2012.

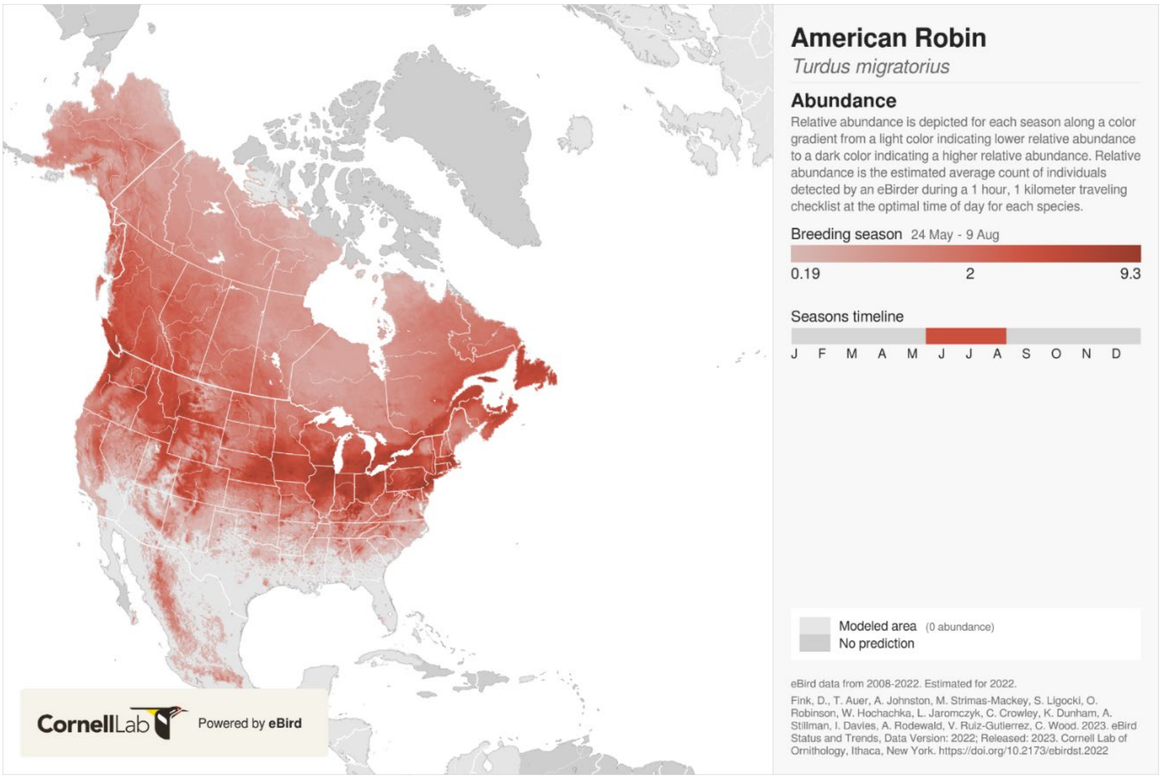

Figure S4. This map shows the distribution of the breeding population of *Turdus migratorius*, the American robin, during the breeding season from 24 May to 9 August. The continental scale dispersion of these birds is amazing, into Alaska and sub-Arctic Canada, and northern Quebec. In the critical areas in the northern part of the mid-continent, e.g. North and South Dakota, their relative abundance is relatively low as compared to Illinois and states further east. Visualization was downloaded from eBird website https://ebird.org/science/status-and-trends (Fink et al., 2023).

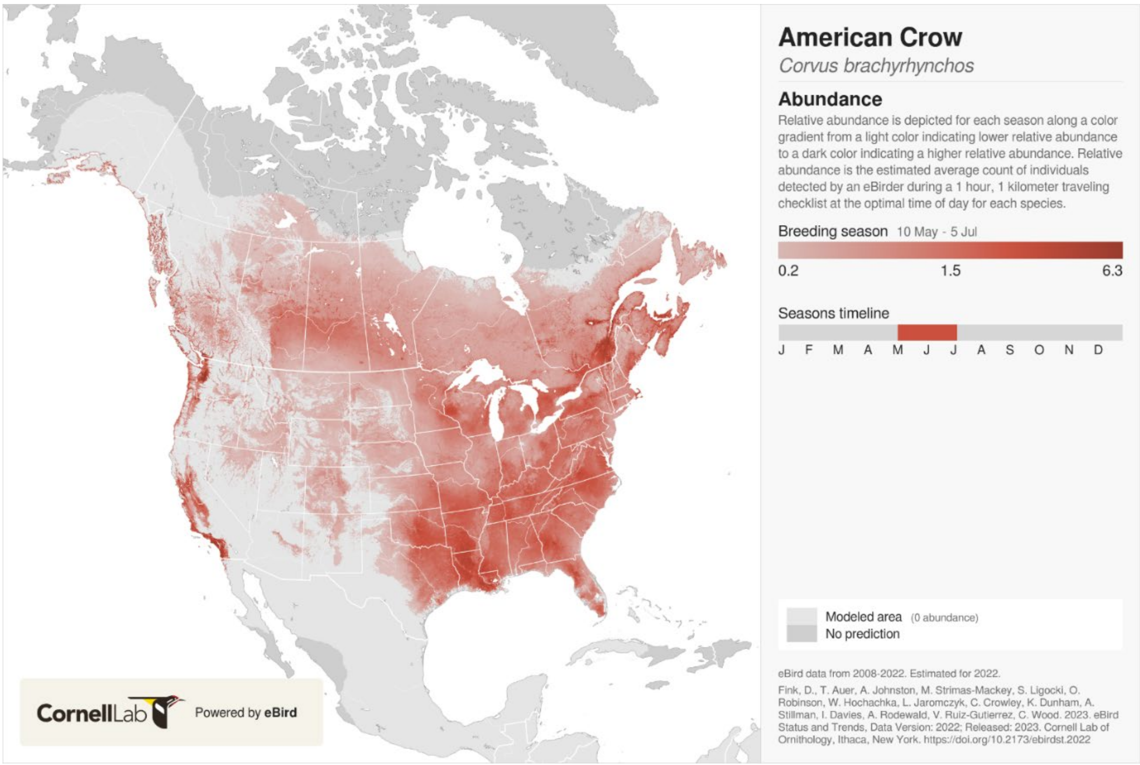

Figure S5. This map shows the distribution of the breeding population of *Corvus brachyrhynchos*, the American crow, during the breeding season from 10 May to 5 July. In the northern portion of the mid-content where cases of WNV have been prevalent (e.g., southern Saskatchewan, North Dakota, South Dakota, Nebraska, and Iowa), the relative abundance of crows is relatively low. Visualization was downloaded from eBird website https://ebird.org/science/status-and-trends (Fink et al., 2023).

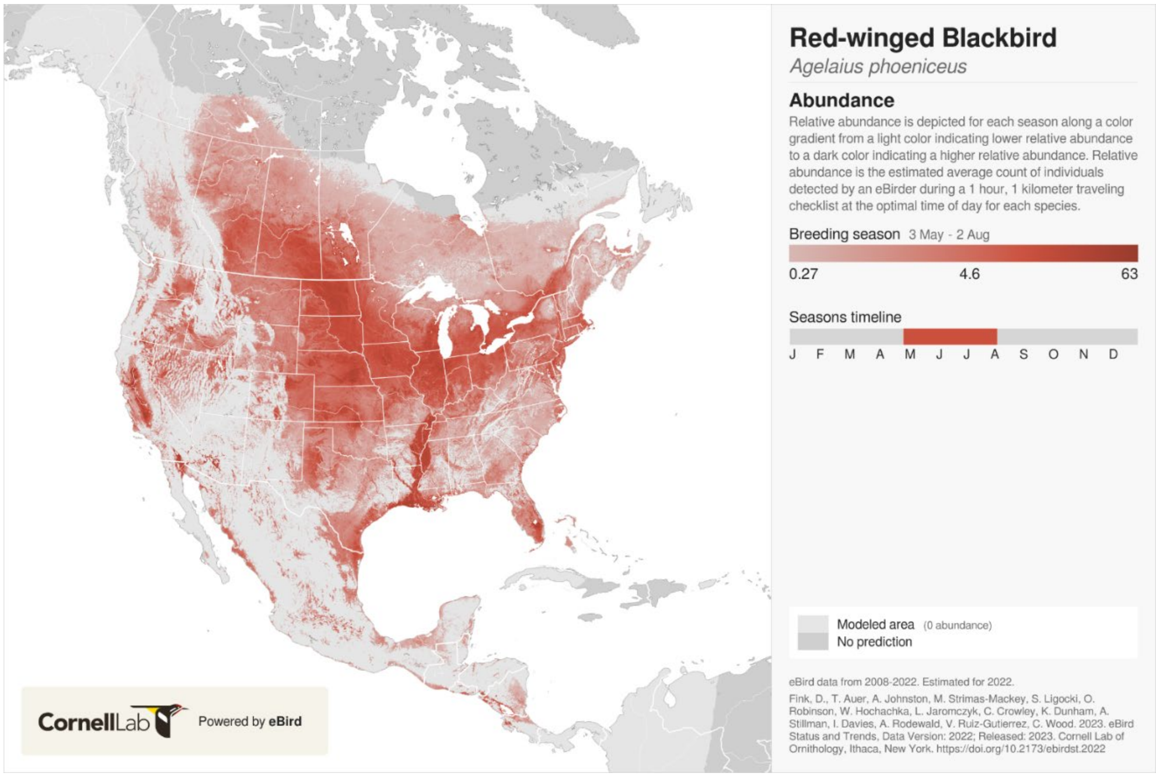

Figure S6. This map shows the distribution of the breeding population of *Agelaius phoeniceus*, red-winged blackbirds during the breeding season from 31 May to 2 August. These birds are prevalent in the northern portion of the mid-content where the incidences of WNV cases have been large (e.g., southern Saskatchewan, North Dakota, South Dakota, and Nebraska). They are also relatively abundant in Illinois, Indiana, Ohio, and eastern Wisconsin where WNV cases are relatively low. Visualization was downloaded from eBird website https://ebird.org/science/status-and-trends (Fink et al., 2023).

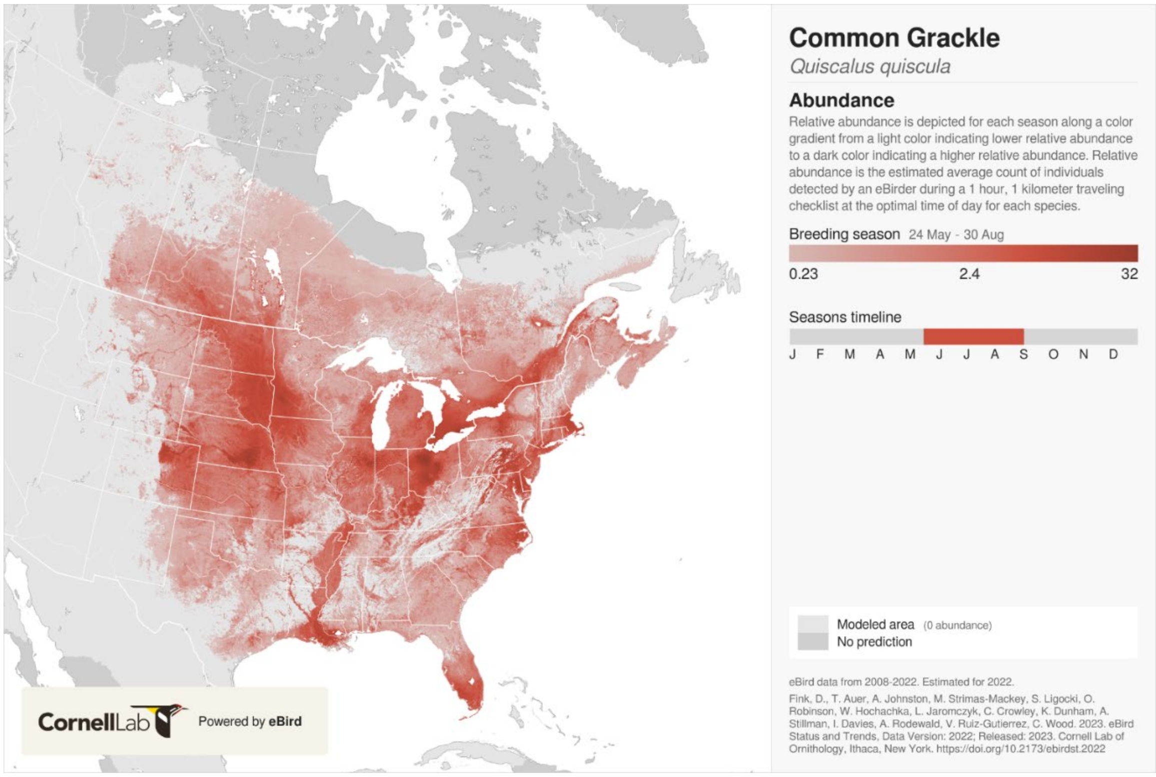

Figure S7. The common grackle (*Quiscalus quiscula*) during breeding season, 24 May to 30 August, is present east of the Rocky Mountains in the U.S. and Canada. Except for local areas along the Mississippi River and southern Florida, abundances in southern states are relatively low. It is relatively abundant in the Northern Great Plains, areas with highest WNV incidences. Visualization was downloaded from the eBird website https://ebird.org/science/status-and-trends (Fink et al., 2023).

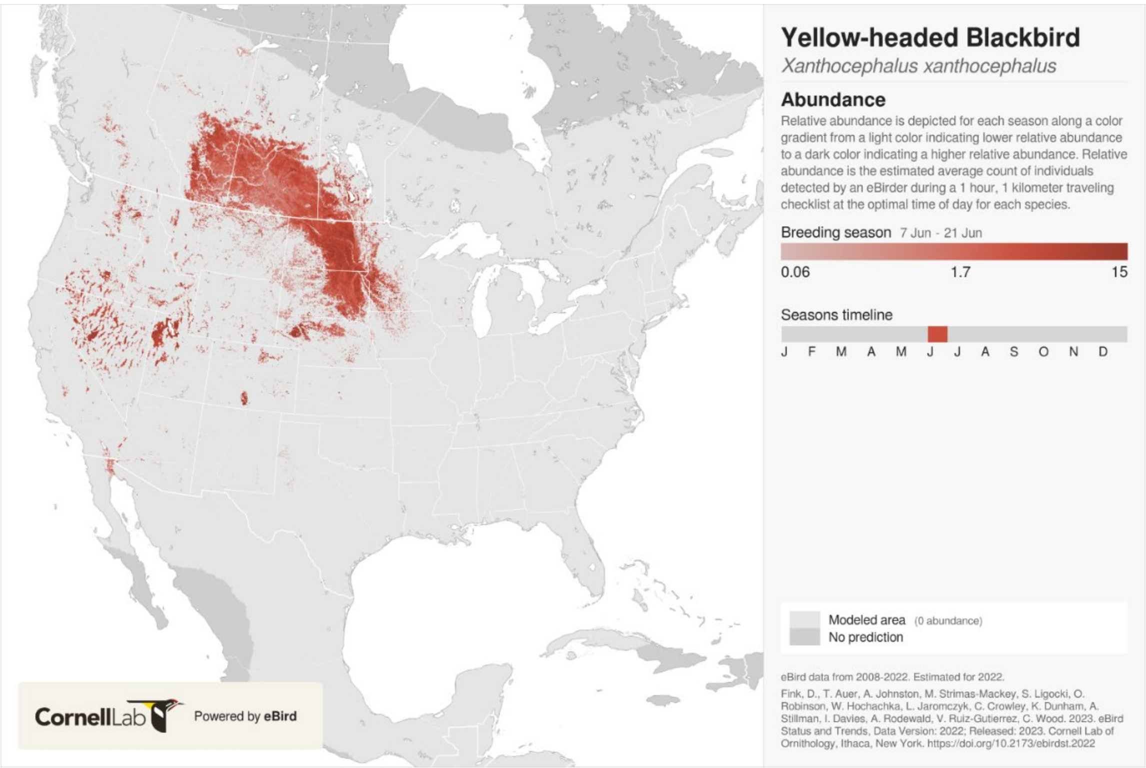

Figure S8. During breeding season, the distribution of the yellow-headed blackbird (*Xanthocephalus xanthocephalus*) is mostly restricted to a wetland terrain known as the "the Prairie Pothole" region (Liu et al., 2011). Almost the entire population covers an area that coincides with highest averages annual incidences of WNV per 100,000 people in North America. Visualization was downloaded from eBird website https://ebird.org/science/status-and-trends (Fink et al., 2023).
